# Facile and Efficient Syntheses of a Series of *N*-Benzyl and *N*-Biphenylmethyl Substituted Imidazole Derivatives Based on (*E*)-Urocanic acid, as Angiotensin II AT1 Receptor Blockers

**DOI:** 10.3390/molecules18077510

**Published:** 2013-06-27

**Authors:** George Agelis, Konstantinos Kelaidonis, Amalia Resvani, Dimitra Kalavrizioti, Maria-Eleni Androutsou, Panagiotis Plotas, Demetrios Vlahakos, Catherine Koukoulitsa, Theodore Tselios, Thomas Mavromoustakos, John Matsoukas

**Affiliations:** 1 Department of Chemistry, University of Patras, Patras 26500, Greece; 2 Eldrug S.A., Patras Science Park, Patras 26504, Greece; 3 Department of Pharmacology, School of Medicine, University of Patras, Patras 26500, Greece; 4 Department of Internal Medicine, ‘ATTIKON’ University Hospital, Athens 12462, Greece; 5 Department of Chemistry, University of Athens, Athens 15771, Greece

**Keywords:** synthesis, AT1 receptor blockers, (*E*)-urocanic acid, *N*-alkylation, docking studies

## Abstract

In the present work, a facile and efficient route for the synthesis of a series of *N*-substituted imidazole derivatives is described. Docking studies have revealed that *N*-substituted imidazole derivatives based on (*E*)-urocanic acid may be potential antihypertensive leads. Therefore, new AT1 receptor blockers bearing either the benzyl or the biphenylmethyl moiety at the *N*-1 or *N*-3 position, either the (*E*)-acrylate or the propanoate fragment and their related acids at the *C*-4 position as well as a halogen atom at the *C*-5 position of the imidazole ring, were synthesized. The newly synthesized analogues were evaluated for binding to human AT1 receptor. The biological results showed that this class of molecules possesses moderate or no activity, thus not always confirming high docking scores. Nonetheless, important conclusions can be derived for their molecular basis of their mode of action and help medicinal chemists to design and synthesize more potent ones. An aliphatic group as in losartan seems to be important for enhancing binding affinity and activity.

## 1. Introduction

**A**ngiotensin II (ANG II) is the octapeptide produced by the Renin-Angiotensin System (RAS) which plays a key role in the pathophysiology of hypertension [1,2,3,4,5]. Inhibitors of the three active sites of RAS have proven to be effective for the treatment of hypertension and congestive heart failure. Research efforts over the last decades have focused on the development of highly selective ANG II AT1 receptor blockers (ARBs) [6]. The DuPont group was the first to develop losartan (DuP 753), an orally effective angiotensin receptor blocker, which is metabolized *in vivo* to the more potent full antagonist EXP 3174 [7,8]. The discovery of losartan has stimulated the design of a large number of congeners [4,5]. Among them, eprosartan, irbesartan, candesartan, valsartan, olmesartan and azilsartan have been launched in the market [6,9,10,11]. Extensive Structure-Activity Relationships (SAR) and pharmacophore modeling studies [12] of ARBs, as well as the available data from literature, have illustrated the key elements required for the design of potent AT1 blockers [1]. Lipophilic substituents, such as the biphenylmethyl fragment substituted with an acidic moiety (tetrazole group, CO_2_H) at the *N*-1 position of a heterocyclic ring and a linear alkyl group, providing an interaction with a hydrophobic pocket of the receptor, are required for potent antagonistic activity [6,13,14,15,16,17]. The DuPont group recommended a lipophilic and electron-withdrawing group such as a halogen atom, CF_3_, ethyl or pentafluoroethyl substituents at the *C*-4 of the imidazole ring and a small sized group such as CH_2_OH or CO_2_H at the *C*-5 capable of forming a hydrogen bond [6,9,18].

Our recent work on the synthesis of AT1 receptor antagonists [18,19] indicated that 4(5)-butylimidazole-based analogues displayed significant antihypertensive activity. As a continuation of our studies [18,19,20], we report herein on the preparation of (*E*)-urocanic acid-based analogues, focusing our attention on the structural modifications on the imidazole ring which would possibly enhance potency. Consequently, we have designed using docking studies and synthesized a series of (*E*)-urocanic acid derivatives bearing the benzyl and the biphenylmethyl tetrazole moiety at the *N*-1 or *N*-3 position of the imidazole ring. Furthermore, these analogues bear the (*E*)-acrylic acid chain of (*E*)-urocanic acid as well as the corresponding saturated side chain at the *C*-4, mimicking the carboxy-terminal region of the octapeptide ANG II [21,22] and a bulky lipophilic and electron-withdrawing group such as a halogen atom at the *C*-5 of the imidazole ring [6,9]. Additionally, some ARBs possessing two acidic groups, such as the tetrazole and the carboxyl group, have exhibited low oral bioavailability (BA) because of their highly polar character [6]. Thus, the rigid acrylic or the saturated acid side chain was masked by esterification resulting in the methyl ester or the bulky ester group (5-methyl-2-oxo-1,3-dioxol-4-yl)methyl or medoxomil [5,9], which is metabolized *in vivo* to the carboxyl moiety and may prove to be an effective structural element, emerging to compounds with improved BA. Finally, the reason for the shortening of the biphenyl group was to evaluate the ability of the tetrazole group to trigger activity located on a phenyl group instead of a biphenyl moiety and on the other hand to examine the ability of a single phenyl group to interact appropriately with the receptor [23]. Our synthetic approach included fast, efficient and regioselective reactions in high yields, allowing the facile introduction of the substituents on the imidazole nucleus. The synthesized analogues were finally tested for their AT1 receptor affinity using binding assays.

## 2. Results and Discussion

### 2.1. Chemistry

**Scheme 1:**
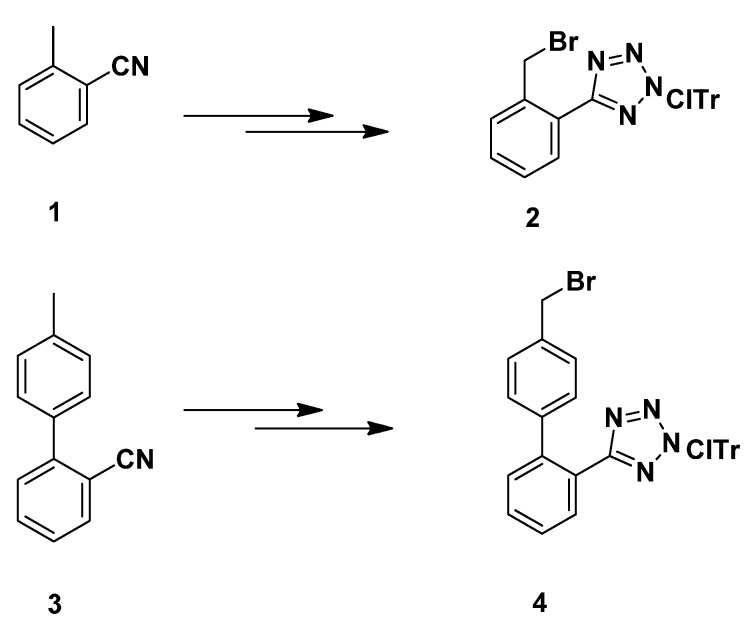
Synthesis of the alkylating agents **2** and **4** [8,18,24,25].

The intermediates **2** and **4** that were used to introduce the benzyl and the biphenylmethyl moiety to the imidazole ring were obtained according to reported methods as outlined in Scheme 1 [8,18,24,25]. In this case, protection of the tetrazole ring with the 2-chlorotrityl group by treatment with 2-chlorotrityl chloride (ClTr-Cl), followed by benzylic bromination provided the requisite alkylating agents **2** and **4** [18].

**Scheme 2:**
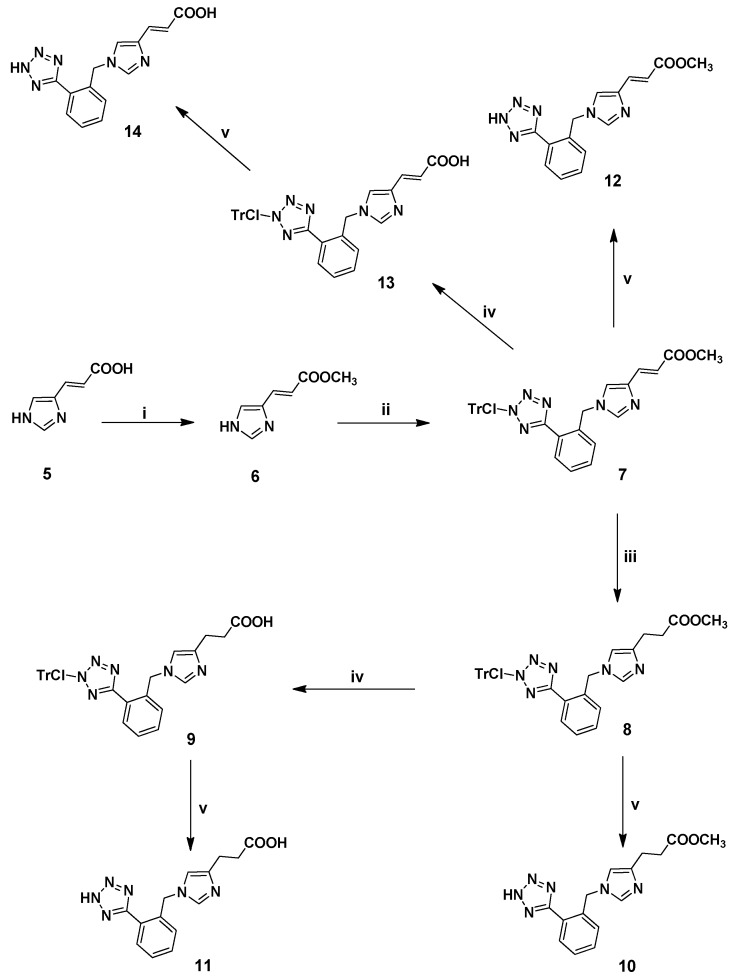
Synthesis of the target *N*-benzyl analogues **10**, **11**, **12**, **14** **^a^**.

The preparation of *N*-benzyl imidazole derivatives **10**–**12** and **14** is depicted in Scheme 2. (*E*)-Urocanic acid (**5**) was converted to the corresponding methyl ester **6** by esterification in dry methanol (MeOH) [20,26]. The ^1^H-NMR spectrum of **6** showed two singlet peaks at *δ* 7.78 and 7.43 ppm corresponding to the H-2 and H-5 of the imidazole ring, respectively. Additionally, the two vinylic protons appeared as doublets at *δ* 6.44 and 7.61 (*J* = 16.0 Hz), respectively and the methoxy group at 3.78 ppm. Alkylation of the methyl ester **6** at the *N*-1 position of the imidazole ring with the benzyl alkylating agent **2**, in the presence of sodium hydride (NaH) in dry *N,N*-dimethylformamide (DMF), afforded **7** in 74% yield. The ^1^H-NMR spectrum of **7** showed a singlet peak at *δ* 5.38 assigned to the methylene protons. Catalytic hydrogenation (Pd/C) of the latter afforded the saturated derivative **8** in excellent yield (90%). The ^1^H-NMR spectra of **8** showed two triplet peaks at *δ* 2.76 and 2.56 (*J* = 7.2 Hz) corresponding to the methylene protons of the saturated side chain. Alkaline hydrolysis [9] of the methyl esters **7** and **8** under mild conditions using a mixture of KOH in 1:1 H_2_O/dioxane, led to the corresponding acids **13** and **9**, respectively. Removal of the ClTr group by treatment with 30% trifluoroacetic acid (TFA) in dichloromethane (CH_2_Cl_2_), in the presence of triethylsilane (Et_3_SiH) as scavenger, provided the final analogues **10**–**12** and **14**.

**^a^** *Reagents and conditions*: (i) MeOH, anhydrous Na_2_SO_4_, conc. H_2_SO_4_, reflux, 24 h; (ii) **2**, NaH (powdered 95%), dry DMF, 0 °C to rt, 4 h; (iii) H_2_, 10% Pd-C, MeOH, rt, 3 h; (iv) KOH, H_2_O/dioxane (1:1), rt, 3 h; (v) 30% TFA in CH_2_Cl_2_, Et_3_SiH, rt, 1 h.

**Scheme 3:**
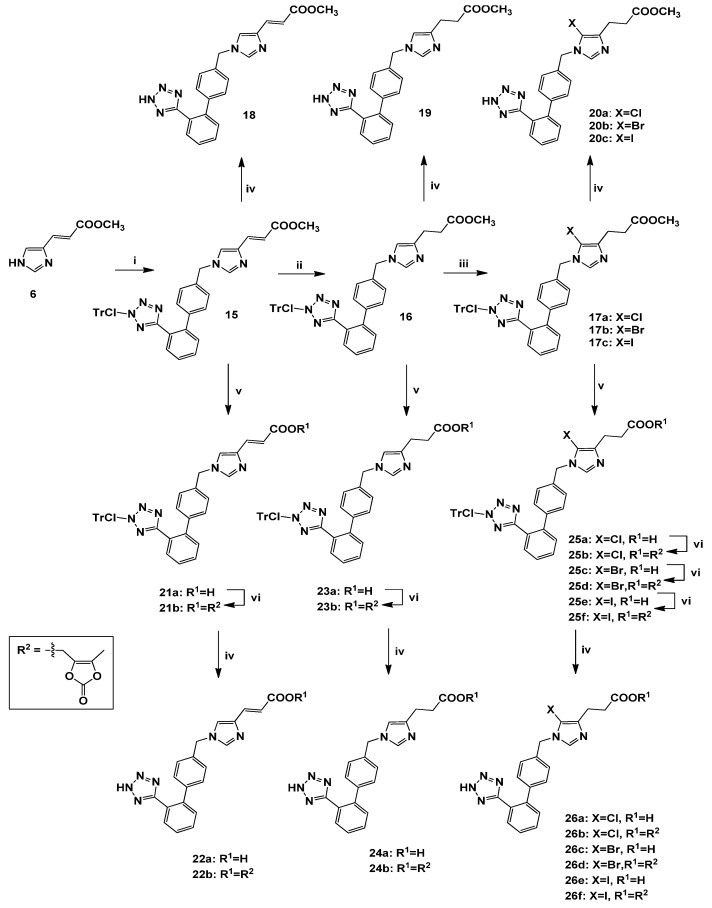
Synthesis of the final *N*-biphenylmethyl analogues **18**, **19**, **20a**–**c**, **22a**–**b**, **24a**–**b**, **26a**–**f ^a^**.

The synthesis of the *N*-biphenylmethyl imidazole derivatives **18**–**20a**–**c**, **22a**–**b**, **24a**–**b** and **26a**–**f** is demonstrated in Scheme 3. Likewise, direct alkylation of the methyl ester **6** at the *N*-1 position of the imidazole ring with the biphenylmethyl alkylating agent **4** afforded the 1,4-disubstituted analogue **15**. The ^1^H-NMR data of **15** showed a singlet peak at *δ* 4.95 due to the methylene protons of the alkylating moiety. Subsequently, hydrogenation of **15** in the presence of 10% Pd-C as catalyst in MeOH, led to the intermediate **16**. Halogenation of **16** at the *C*-5 position of the imidazole ring with the appropriate *N*-halosuccinimide (NXS, X = Cl, Br, I) [18], afforded the halogenated derivatives **17a**–**c**. The ^1^H-NMR spectra showed the absence of the H-5 signal of the imidazole ring at 6.47 ppm appearing in **16**. Saponification of the methyl esters **15**–**17a**–**c** was mediated by an aqueous solution of KOH in dioxane for 3 h at rt, to afford the corresponding acids **21a**, **23a**, **25a**, **25c** and **25e**, respectively. The ^1^H-NMR spectra confirmed the absence of the methoxy group at 3.56–3.77 ppm. Treatment of the latter acids with medoxomil chloride (4-chloromethyl-5-methyl-2-oxo-1,3-dioxole) in the presence of potassium carbonate (K_2_CO_3_) in dry *N,N*-dimethylacetamide (DMA), [9] furnished the esters **21b**, **23b**, **25b**, **25d** and **25f**. The presence of the -OCH_2_ protons signal at 5.02–4.71 ppm as well as the methyl protons signal at 2.02–2.16, unequivocally confirmed the introduction of the medoxomil group. Detritylation of the tetrazole group was accomplished by treatment with TFA in CH_2_Cl_2_, resulting in the target compounds **18**–**20a**–**c**, **22a**–**b**, **24a**–**b** and **26a**–**f**.

***^a^*** *Reagents and conditions*: (i) **4**, NaH (powdered 95%), dry DMF, 0 °C to rt, 4 h; (ii) H_2_, 10% Pd-C, MeOH, rt, 3 h; (iii) NXS, X = Br, I in dry DMF or NCS in MeCN, 0 °C to rt, 4 h; (iv) 30% TFA in CH_2_Cl_2_, Et_3_SiH, rt, 1 h; (v) KOH, H_2_O/dioxane (1:1), rt, 3 h; (vi) K_2_CO_3_, dry DMA, 4-chloromethyl-5-methyl-2-oxo-1,3-dioxole, rt, 4 h.

**Scheme 4:**
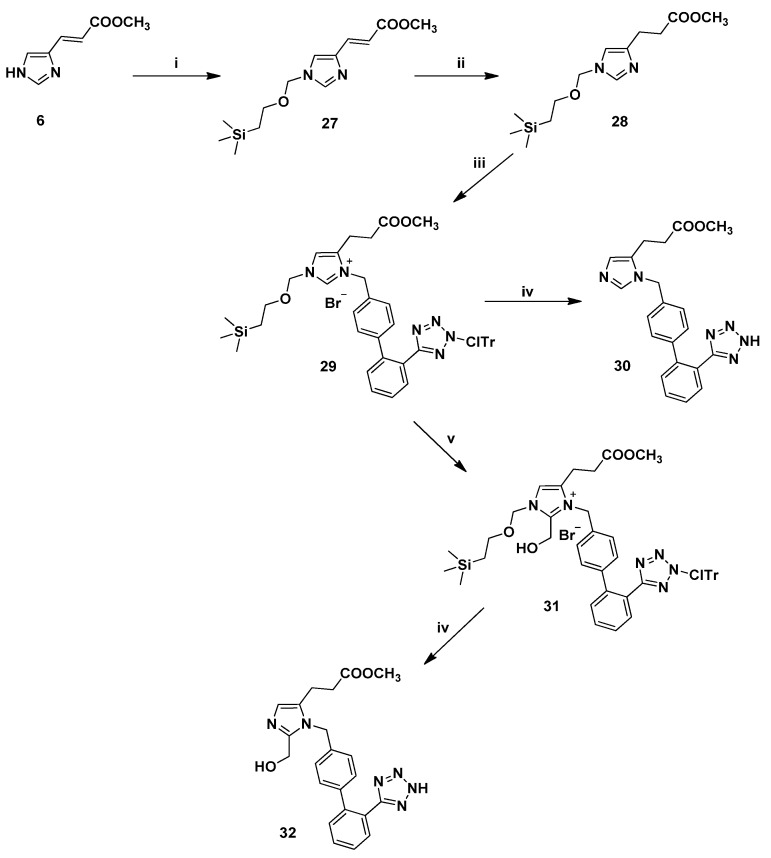
Synthesis of the final *N*-biphenylmethyl substituted analogues **30** and **32** ^a^.

^a^ *Reagents and conditions*: (i) SEM-Cl, NaH (powdered 95%), dry DMF, 0 °C to rt, 2 h; (ii) H_2_, 10% Pd-C, MeOH, rt, 3 h; (iii) **4**, CH_2_Cl_2_, reflux, 3 h; (iv) 30% TFA in CH_2_Cl_2_, Et_3_SiH, rt, 1 h; (v) 37% formalin, diisopropylethylamine, DMF, 85 °C, 1 h.

Finally, the preparation of the *N*-biphenylmethyl imidazole derivatives **30** and **32** is depicted in Scheme 4. Firstly, the imidazole ring was protected at the *N*-1 by the 2-(trimethylsilyl)ethoxymethyl (SEM) group using standard conditions [18,27,28]. Thus, treatment of the unsaturated methyl ester **6** with SEM-Cl in the presence of NaH in dry DMF, at ambient temperature for 2 h, led to **27** in 78% yield. It is worth noting, that using the latter reaction conditions only the desired 1,4-regioisomer was formed, as indicated by HPLC and ^1^H-NMR. 

The resulting derivative **27** was subjected to hydrogenation in the presence of catalyst 10% Pd-C in MeOH to afford **28**, in 91% yield. Regioselective alkylation at the *N*-3 position was performed in the presence of the alkylating reagent **4** in CH_2_Cl_2_ under reflux for 3 h, resulting in the intermediate salt **29** in high yield (81%). Thus, the SEM group was proven to be an excellent choice for the protection of the *N*-1 followed by regioselective alkylation at the *N*-3 of the imidazole ring. At this point, we were ready to perform the introduction of the hydroxymethyl group at the *C*-2 of the imidazole ring of the alkylated analogue **29**. According to our strategy [18], the hydroxymethylation was promptly carried out in a sealed tube by treatment with diisopropylethylamine and 37% formalin in DMF at 85 °C for 1 h. The obtained residue was purified by column chromatography to afford the hydroxymethylated product **31** in excellent yield (91%) and purity. The ^1^H-NMR spectrum of **31** showed the presence of a singlet peak at 4.72 ppm due to the hydroxymethyl protons. Removal of the ClTr group by means of 30% TFA in CH_2_Cl_2_ and Et_3_SiH led to the 1,5-disubstituted imidazole analogues **30** and **32**.

### 2.2. Pharmacology

The new synthesized analogues were evaluated in radioligand binding assay at a final concentration of 10^−5^ M. Although the used concentration was high enough, there were indications for moderate activity of the analogues **12** and **18**. Competitive binding experiments revealed that the latter analogues caused 40.1% and 59.4% displacement of [^125^I]-Sar^1^-Ile^8^-ANG II from the AT1 receptor, respectively, whereas losartan at the same conditions caused 100% displacement.

### 2.3. Docking Studies

The synthesized analogues have been rationalized based on their highest docking scores (Table 1). We notice that some of these analogues show higher scoring than losartan as reported in our previous paper [18].

**Table 1:** Highest Docking Scores of the synthesized analogues obtained with GLIDE/IFD ^a^.

Compounds	Docking Score
Losartan	−12.114
**10**	−9.625
**12**	−10.276
**18**	−12.089
**19**	−11.395
**20a**	−10.397
**20b**	−11.099
**20c**	−11.899
**22a**	−11.577
**22b**	−14.401
**24a**	−12.321
**24b**	−13.570
**26a**	−10.577
**26b**	−13.463
**26c**	−9.404
**26d**	−13.047
**26e**	−13.168
**26f**	−12.017
**30**	−12.281
**32**	−11.012

^a^ Induced Fit Docking/XP module.

**Figure 1:**
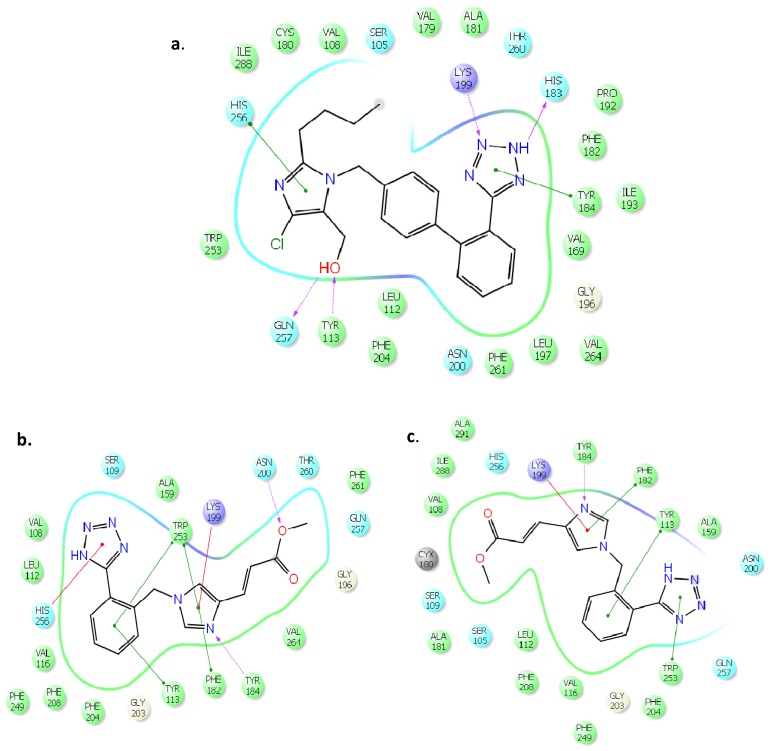

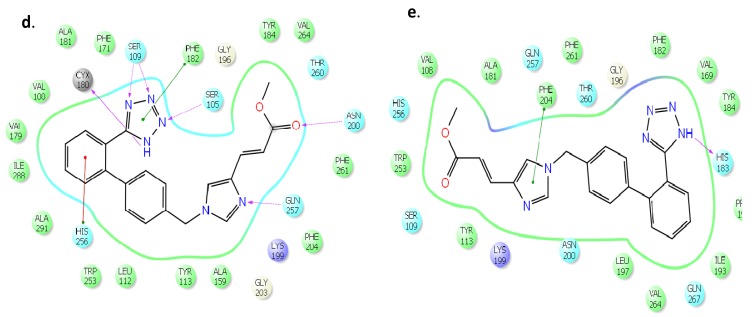
Ligand interactions of **a** losartan; **b** and **c** analogue **12**; **d** and **e** analogue **18** with the aminoacids of the active site of AT1; (**b** and **d** represent orientations of analogues **12** and **18** with the highest scoring and **c** and **e.** represent orientations with low scoring).

**Figure 2:**
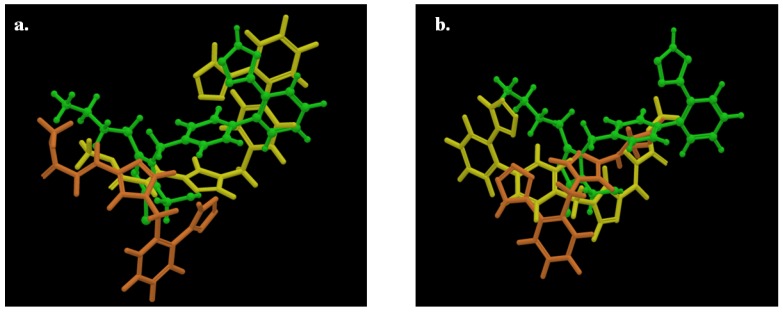
**a.** Superimposition of losartan (green), with low scoring orientations of analogues **12** (orange) and **18** (yellow). **b.** with the highest scoring orientations.

However, high scores could not rationalize the pharmacological results which showed that most of the synthesized analogues were inactive and only few of them showed moderate activity. In order to comprehend the pharmacological data, we have used as a template for comparison the putative bioactive conformation of losartan in the AT1 receptor presented by the pose of Figure 1a. Interestingly, the inactive compounds adopted losartan’s orientation with poses of low scoring. The highest scoring orientations differed from that of losartan (Figure 1). As a result of this, the inactive compounds, even though docked in the same cavity, exerted different critical interactions that explain their inability to possess pharmacological activity. This is also applied with analogues **12** and **18** that showed 40.1% and 59.4% displacement of [^125^I]-Sar^1^-Ile^8^-ANG II from the AT1 receptor (Figure 2). 

As it is shown in Figure 1b,d the poses that showed the highest scoring (**12**, −10.276 kcal/mol and **18**, −12.089 kcal/mol), adopted orientations that did not match that of losartan. For example, analogue **18** is lacking the hydrogen bonding with Lys199 and both **12** and **18** are lacking the hydrogen bonding with His183, Gln257 and Tyr113. However, the poses that resembled the orientation of losartan (Figure 1c,e) showed low scorings (**12**, −8.779 kcal/mol and **18**, −5.217 kcal/mol). This is attributed to the fact that both **12** and **18** cannot adopt the maximal critical interactions. For example, **18** forms only two hydrogen bondings (losartan forms four) and **12** only one. It appears that docking experiments could shed light on the required molecular interactions for drug activity only when pharmacological data were obtained.

## 3. Experimental

### 3.1. General

Starting materials were purchased by Aldrich (Patras, Greece) and used as received**.** Hydrogenation reaction was carried out in a Parr hydrogenation apparatus equipped with a 4 L hydrogen tank. The ^1^H-NMR and ^13^C-NMR spectra were recorded on a Bruker Avance DPX spectrometer at 400.13 MHz and 161.76 MHz, respectively. Chemical shifts are given in *δ* values (ppm) using tetramethylsilane as the internal standard and coupling constants (*J*) are given in Hertz (Hz). HPLC analysis was performed on an Alliance Waters 2695 equipped with a Waters 2996 Photodiode Array Detector UV-Vis, using the XBridge Waters C18 column (4.6 × 150 mm, 3.5 μm) as stationary phase and a gradient of H_2_O/MeCN both containing 0.08% TFA as mobile phase. Electrospray-ionization mass spectra (ESI-MS) were obtained on a UPLC (ultra performance liquid chromatography) equipped with SQ detector Acquity^TM^ by Waters. All reactions were carried out in anhydrous solvents. Analytical TLC was performed on silica gel 60 F_254_ plates (Merck, Germany) and visualized by UV irradiation and iodine. Silica gel 60N (particle size 0.04–0.063 mm) was used for column chomatography.

### 3.2. Synthesis

#### 3.2.1. General Procedure 1: Alkylation of the (*E*)-urocanic Methyl Esters at the Ν-1 Position

To a solution of **6** (2.0 g, 13.16 mmol) in dry DMF (25 mL), dry ΝaH (powdered 95%, 0.35 g, 14.47 mmol) was added and the resulting suspension was stirred for 30 min at 0 °C under nitrogen Then, **4** (8.56 g, 14.48 mmol) was added in two portions and the mixture was stirred for 4 h at RT. The mixture was diluted in H_2_O, extracted with CH_2_Cl_2_ and the organic phase was washed successively with 5% w/v citric acid, brine, dried over Na_2_SO_4_ and concentrated. The residue was purified by flash column chromatography (7:3 EtOAc:hexanes) to afford **15**.

#### 3.2.2. General Procedure 2: Catalytic Hydrogenation of the *Ν*-1 Alkylated (*E*)-Urocanic methyl Esters

A mixture of **15** (5.0 g, 7.54 mmol), 10% w/w Pd-C (0.50 g) in MeOH (20.0 mL) was stirred under a hydrogen atmosphere (3 bar) at ambient temperature for 3 h. The catalyst was filtered off by a Celite pad and the filtrate was concentrated *in vacuo*. The residue was purified by flash column chromatography (EtOAc) to provide **16**.

#### 3.2.3. General Procedure 3: Halogenation of the *Ν*-1 Alkylated Imidazole Derivatives

To a solution of **16** (1.0 g, 1.5 mmol) in dry DMF (5.0 mL) at 0 °C under nitrogen, NBS (0.29 g, 1.65 mmol) was added in three portions and the mixture was allowed to cool at room temperature. After 4 h, the solvent was removed and the residue was purified by flash column chromatography (8:2, EtOAc:hexanes) to provide **17b**.

#### 3.2.4. General Procedure 4: Alkaline Hydrolysis of the *Ν*-Substituted Imidazole Methyl Esters

To a solution of **16** (2.50 g, 3.75 mmol) in H_2_O/dioxane (10.0 mL, 1:1) was added fine powdered ΚΟΗ (2.10 g, 37.50 mmol) and the resulting mixture was stirred at ambient temperature for 3 h. The dioxane was removed by distillation *in vacuo* and to the residual solution was added 1 N HCl to give a white precipitate **23a** which was collected by vacuum filtration.

#### 3.2.5. General Procedure 5: Esterification of the *Ν*-Substituted Imidazole Carboxylic Acids with the (5-Methyl-2-oxo-1,3-dioxol-4-yl)methyl or Medoxomil Group

To a solution of **23a** (2.0 g, 3.07 mmol) in dry DMA (5.0 mL) under nitrogen was added K_2_CO_3_ (0.88 g, 6.41 mmol) and the mixture was stirred at ambient temperature for 30 min. A solution of 4-chloromethyl-5-methyl-2-oxo-1,3-dioxole (0.67 g, 4.56 mmol) in dry DMA was added dropwise and the resulting mixture was stirred for 4 h. Then, the mixture was diluted with EtOAc and the organic phase was washed with H_2_O, brine, dried over Na_2_SO_4_ and concentrated *in vacuo*. The crude product was purified by flash column chromatography (8:2 EtOAc:hexanes) to afford **23b**.

#### 3.2.6. General Procedure 6: Removal of the 2-Chlorotrityl Protecting Group

To a solution of **16** (0.50 g, 0.75 mmol) in 30% TFA in CH_2_Cl_2_ (2.0 mL), TES (0.10 mL, 0.63 mmol) was added dropwise and the resulting solution was stirred for 1 h at ambient temperature. The reaction mixture was concentrated and recrystallized from diethyl ether to afford **19**. 

#### 3.2.7. Synthesis of 1,4-Disubstituted Benzyl Analogues **10**, **11**, **12**, **14**

*N-(2-Chlorotrityl)-5-(2-bromobenzyl)tetrazole* (**2**)*.* Prepared from **1** according to the literature method [8,18,24,25]. Yield 78%; R*_f_* = 0.46 (2:8 EtOAc:hexanes); ESI-MS (*m/z*): 238.27 (M+H^+^-ClTr), 277.78 (ClTr); ^1^H-NMR (CDCl_3_): *δ* 8.22–8.18 (m, 1H), 7.51–7.32 (m, 12H), 7.25–7.21 (m, 4H), 6.86 (d, 1H, *J* = 7.6 Hz), 4.92 (s, 2H); ^13^C-NMR (CDCl_3_): *δ* 162.90, 141.28, 136.87, 131.64, 130.46, 129.96, 128.94, 128.42, 127.96, 127.84, 127.29, 126.43, 83.45, 32.42. Anal. Calcd for C_27_H_20_N_4_ClBr (%): C: 62.87; H: 3.91; N: 10.86. Found (%): C: 62.99; H: 4.00; N: 10.54.

*Methyl 3-(1H-imidazol-4-yl)acrylate* (**6**)*.* Prepared from **5** according to the literature method [20,26]. Yield 95%; M.p. 92–94 °C; R*_f_* = 0.42 (9:1 CHCl_3_:MeOH); ESI-MS (*m/z*): 153.22 (M+H^+^); ^1^Η-ΝΜR (CD_3_OD)**:** *δ* 7.78 (s, 1H), 7.61 (d, 1Η, *J* = 16.0 Hz), 7.43 (s, 1H), 6.44 (d, 1H, *J* = 16.0 Hz), 3.78 (s, 3H); ^13^C-NMR (CDCl_3_): *δ* 168.14, 137.43, 135.25, 134.35, 124.0, 114.35, 50.61. Anal. Calcd for C_7_H_8_N_2_O_2_ (%): C: 55.26; H: 5.30; N: 18.41. Found (%): C: 55.21; H: 5.39; N: 18.37. All data were consistent with literature [26].

*(E)-Methyl 1-[[1-[[N-(2-chlorotrityl)]-1H-tetrazol-5-yl]phenyl-2-yl]methyl]imidazole-4-acrylate* (**7**). General procedure 1 was employed for the preparation of **7** using **2** as alkylating agent. Yield 74%; R*_f_* = 0.38 (8:2 EtOAc:hexanes); ESI-MS (*m/z*): 587.27 (M+H^+^), 309.37 (M+H^+^-ClTr), 277.88 (ClTr); ^1^Η-ΝΜR (CDCl_3_): *δ* 8.28 (dd, 1H, *J* = 2.0, 7.6 Hz), 7.53–7.10 (m, 19H), 6.76 (s, 1H), 6.48 (d, 1H, *J* = 15.6 Hz), 5.38 (s, 2H), 3.77 (s, 3H). Anal. Calcd for C_34_H_27_N_6_O_2_Cl (%): C: 74.02; H: 4.30; N: 13.28. Found (%): C: 73.96; H: 4.22; N: 13.32.

*Methyl 1-[[1-[[N-(2-chlorotrityl)]-1H-tetrazol-5-yl]phenyl-2-yl]methyl]imidazole-4-propanoate* (**8**). General procedure 2 was employed for the preparation of **8**. Yield 90%; R*_f_* = 0.45 (9:1 CHCl_3_:MeOH); ESI-MS (*m/z*): 589.17 (M+H^+^), 311.45 (M+H^+^-ClTr), 277.38 (ClTr); ^1^Η-ΝΜR (CDCl_3_): *δ* 8.18–8.15 (m, 1H), 7.42–7.26 (m, 9H), 7.07–6.99 (m, 9H), 6.37 (s, 1H), 5.27 (s, 2H), 3.57 (s, 3H), 2.76 (t, 2H, *J* = 7.2 Hz), 2.56 (t, 2H, *J* = 7.2 Hz). Anal. Calcd for C_34_H_29_N_6_O_2_Cl (%): C: 69.32; H: 4.96; N: 14.27. Found (%): C: 69.45; H: 4.88; N: 14.32. 

*1-[[1-[[N-(2-Chlorotrityl)]-1H-tetrazol-5-yl]phenyl-2-yl]methyl]imidazole-4-propanoic acid* (**9**). General procedure 4 was employed for the preparation of **9**. Yield 93%; R*_f_* = 0.31 (8.5:1.5 CHCl_3_:MeOH); ESI-MS (*m/z*): 576.19 (M+H^+^), 299.63 (M+H^+^-ClTr), 277.77 (ClTr); ^1^Η-ΝΜR (CDCl_3_): *δ* 8.31 (d, 1Η, *J* = 7.2 Hz), 7.54–7.11 (m, 17H), 6.85 (d, 1H, *J* = 7.6 Hz), 6.42 (s, 1H), 5.40 (s, 2H), 2.73 (bs, 2H), 2.65 (bs, 2H). Anal. Calcd for C_33_H_27_N_6_O_2_ (%): C: 68.92; H: 4.73; N: 14.61. Found (%): C: 68.92; H: 4.73; N: 14.61.

*Methyl 1-[[1-(1H-tetrazol-5-yl)phenyl-2-yl]methyl]imidazole-4-propanoate* (**10**). General procedure 6 was employed for the preparation of **10**. Yield 91%; R*_f_* = 0.48 (8:2 CHCl_3_:MeOH); ESI-MS (*m/z*): 313.34 (M+H^+^); ^1^Η-ΝΜR (CD_3_OD): *δ* 8.80 (s, 1H), 7.96-7.93 (m, 1H), 7.64–7.60 (m, 3H), 7.33 (s, 1Η), 5.75 (s, 2H), 3.65 (s, 3H), 2.94 (t, 2H, *J* = 7.2 Hz), 2.69 (t, 2H, *J* = 7.2 Hz). Anal. Calcd for C_15_H_16_N_6_O_2_∙CF_3_COOH (%): C: 47.89; H: 4.02; N: 19.71. Found (%): C: 47.78; H: 3.95; N: 19.87.

*1-[[1-(1H-Tetrazol-5-yl)phenyl-2-yl]methyl]imidazole-4-propanoic acid* (**11**). General procedure 6 was employed for the preparation of **11**. Yield 88%; R*_f_* = 0.37 (4:1:1 *n*-butanol:acetic acid:H_2_O); ESI-MS (*m/z*): 297.24 (M+H^+^); ^1^Η-ΝΜR (CD_3_OD): *δ* 8.83 (s, 1H), 7.97–7.93 (m, 1H), 7.65–7.59 (m, 3H), 7.36 (s, 1H), 5.76 (s, 2H), 2.93 (t, 2H, *J* = 7.2 Hz), 2.66 (t, 2H, *J* = 7.2 Hz). Anal. Calcd for C_14_H_14_N_6_O_2_∙CF_3_COOH (%): C: 46.11; H: 3.67; N: 20.38. Found (%): C: 46.02; H: 3.57; N: 20.48. 

*(E)-Methyl 1-[[1-(1H-tetrazol-5-yl)phenyl-2-yl]methyl]imidazole-4-acrylate* (**12**). General procedure 6 was employed for the preparation of **12**. Yield 95%; R*_f_* = 0.48 (8:2 CHCl_3_:MeOH); ESI-MS (*m/z*): 311.24 (M+H^+^); ^1^Η-ΝΜR (DMSO-*d_6_*): *δ* 8.46 (s, 1H), 7.90 (s, 1H), 7.79 (s, 1H), 7.62 (bs, 2H), 7.58 (m, 4Η), 7.50 (d, 1H, *J* = 15.6 Hz), 7.34 (s, 1H), 6.46 (d, 1H, *J* = 15.6 Hz), 5.68 (s, 2H), 3.78 (s, 3H). Anal. Calcd for C_15_H_14_N_6_O_2_∙CF_3_COOH (%): C: 48.12; H: 3.56; N: 19.81. Found (%): C: 48.22; H: 3.47; N: 19.71.

*(E)-1-[[1-[[N-(2-Chlorotrityl)]-1H-tetrazol-5-yl]phenyl-2-yl]methyl]imidazole-4-acrylic acid* (**13**). General procedure 4 was employed for the preparation of **13**. Yield 95%; R*_f_* = 0.30 (9:1 CHCl_3_:MeOH); ESI-MS (*m/z*): 574.15 (M+H^+^), 297.54 (M+H^+^-ClTr), 277.78 (ClTr); ^1^Η-ΝΜR (CDCl_3_): *δ* 8.28 (d, 2Η, *J* = 5.6 Hz), 7.52–7.23 (m, 12H), 7.10 (d, 6H, *J* = 5.6 Hz), 6.74 (bs, 1H), 6.44 (d, 1H, *J* = 15.6 Hz), 5.36 (s, 2H). Anal. Calcd for C_33_H_25_N_6_O_2_Cl (%): C: 69.17; H: 4.40; N: 14.67. Found (%): C: 69.11; H: 4.29; N: 14.72.

*(E)-1-[[1-(1H-Tetrazol-5-yl)phenyl-2-yl]methyl]imidazole-4-acrylic acid* (**14**). General procedure 6 was employed for the preparation of **14**. Yield 95%; R*_f_* = 0.50 (4:1:1 *n*-butanol:acetic acid:H_2_O); ESI-MS (*m/z*): 297.30 (M+H^+^); ^1^Η-ΝΜR (CD_3_OD): *δ* 8.85 (s, 1H), 7.98–7.95 (m, 1H), 7.84 (s, 1H), 7.72–7.64 (m, 3H), 7.48 (d, 1H, *J* = 16.0 Hz), 6.49 (d, 1H, *J* = 16.0 Hz), 5.80 (s, 2H). Anal. Calcd for C_14_H_12_N_6_O_2_∙CF_3_COOH (%): C: 48.12; H: 3.56; N: 19.81. Found (%): C: 48.22; H: 3.47; N: 19.71.

#### 3.2.8. Synthesis of 1,4-Disubstituted biphenylmethyl analogues **18**, **19**, **20a**–**c**, **22a**–**b**, **24a**–**b**, **26a**–**f**

*Ν-(2-Chlorotrityl)-5-[4′-(bromomethyl)biphenyl-2-yl]tetrazole* (**4**). Prepared from **3** according to the literature method [8,19,25,26]. Yield 80%; M.p. 155–157 °C; R*_f_* = 0.33 (1.5:8.5 EtOAc:hexanes); ESI-MS (*m/z*): 315.48 (M+H^+^-ClTr), 277.22 (ClTr); ^1^Η-ΝΜR (CDCl_3_): *δ* 8.02–7.89 (m, 1H), 7.60–6.87 (m, 20H), 6.79 (dd, 1H, *J* = 1.5, 8.0 Hz), 4.43 (s, 2H); ^13^C-NMR (CDCl_3_): *δ* 163.95, 141.63, 141.47, 140.64, 139.49, 136.34, 132.13, 131.75, 130.79, 130.53, 130.15, 129.69, 128.87, 128.66, 127.97, 127.91, 126.68, 81.96, 33.45. Anal. Calcd for C_33_H_24_Ν_4_ClBr (%): C: 66.96; H: 4.09; N: 9.47. Found (%): C: 66.88; H: 4.15; N: 9.42.

*(E)-Methyl 1-[[2′-[[N-(2-chlorotrityl)]-1H-tetrazol-5-yl]biphenyl-4-yl]methyl]imidazole-4-acrylate* (**15**). General procedure 1 was employed for the preparation of **15** using **4** as alkylating agent. Yield 73%; R*_f_* = 0.50 (EtOAc); ESI-MS (*m/z*): 664.19 (M+H^+^), 387.29 (M+H^+^-ClTr), 277.38 (ClTr); ^1^Η-ΝΜR (CDCl_3_): *δ* 8.28 (dd, 1H, *J* = 1.8, 7.6 Hz), 7.53–7.12 (m, 22H), 6.84 (m, 2H), 6.54 (d, 1H, *J* = 15.6 Hz), 4.95 (s, 2H), 3.77 (s, 3H); ^13^C-NMR (CDCl_3_): *δ* 168.20, 137.43, 162.87, 140.48, 139.14, 138.89, 138.22, 136.33, 134.05, 132.22, 131.84, 130.99, 130.54, 129.29, 128.86, 128.30, 126.97, 126.31, 122.18, 115.53, 82.66, 51.63, 49.53. Anal. Calcd for C_40_H_31_N_6_O_2_Cl (%): C: 72.44; H: 4.71; N: 12.67. Found (%): C: 72.38; H: 4.77; N: 12.62.

*Methyl 1-[[2′-[[N-(2-chlorotrityl)]-1H-tetrazol-5-yl]biphenyl-4-yl]methyl]imidazole-4-propanoate* (**16**). General procedure 2 was employed for the preparation of **16**. Yield 88%; R*_f_* = 0.55 (9:1 CHCl_3_:MeOH); ESI-MS (*m/z*): 666.19 (M+H^+^), 388.39 (M+H^+^-ClTr), 277.77 (ClTr); ^1^Η-ΝΜR (CDCl_3_): *δ* 7.98 (dd, 1H, *J* = 2.0, 7.6 Hz), 7.53–7.12 (m, 14H), 6.89–6.83 (m, 7H), 6.72 (dd, 1H, *J* = 1.2, 8.0 Hz), 6.47 (s, 1H), 4.87 (s, 2H), 3.65 (s, 3H), 2.84 (t, 2H, *J* = 7.6 Hz), 2.64 (t, 2H, *J* = 7.6 Hz). Anal. Calcd for C_40_H_33_N_6_O_2_Cl (%): C: 72.23; H: 5.00; N: 12.63. Found (%): C: 72.18; H: 5.09; N: 12.57. 

*Methyl 5-chloro-1-[[2′-[[N-(2-chlorotrityl)]-1H-tetrazol-5-yl]biphenyl-4-yl]methyl]imidazole-4-propanoate* (**17a**). General procedure 3 was employed for the preparation of **17a** using NCS in MeCN. Yield 85%; R*_f_* = 0.49 (EtOAc); ESI-MS (*m/z*): 700.59 (M+H^+^), 422.86 (M+H^+^-ClTr), 277.77 (ClTr); ^1^Η-ΝΜR (CDCl_3_): *δ* 7.97 (dd, 1Η, *J* = 1.2, 7.2 Hz), 7.52–7.14 (m, 15H), 6.90–6.84 (m, 6H), 6.72 (d, 1H, *J* = 8.0 Hz), 4.89 (s, 2H), 3.68 (s, 3H), 2.87 (t, 2H, *J* = 7.6 Hz), 2.70 (t, 2H, *J* = 7.6 Hz). C_40_H_32_Cl_2_N_6_O_2_. Anal. Calcd for C_40_H_32_N_6_O_2_Cl_2_ (%): C: 68.67; H: 4.61; N: 12.01. Found (%): C: 67.92; H: 4.78; N: 11.84.

*Methyl 5-bromo-1-[[2′-[[N-(2-chlorotrityl)]-1H-tetrazol-5-yl]biphenyl-4-yl]methyl]imidazole-4-propanoate* (**17b**). General procedure 3 was employed for the preparation of **17b** using NBS in DMF. Yield 68%; R*_f_* = 0.47 (EtOAc); ESI-MS (*m/z*): 745.54 (M+H^+^), 467.80 (M+H^+^-ClTr), 277.80 (ClTr); ^1^Η-ΝΜR (CDCl_3_): *δ* 7.84 (dd, 1H, *J* = 1.2, 7.2 Hz ), 7.46-7.24 (m, 8H), 7.18-6.79 (m, 13H), 6.68 (dd, 1H, *J* = 1.6, 8.0 Hz), 4.88 (s, 2H), 3.56 (s, 3H), 2.73 (t, 2H, *J* = 7.6 Hz), 2.58 (t, 2H, *J* = 7.6 Hz). Anal. Calcd for C_40_H_32_N_6_O_2_ClBr (%): C: 64.57; H: 4.33; N: 11.29. Found (%): C: 64.68; H: 4.40; N: 11.23.

*Methyl 5-iodo-1-[[2′-[[N-(2-chlorotrityl)]-1H-tetrazol-5-yl]biphenyl-4-yl]methyl]imidazole-4-propanoate* (**17c**). General procedure 3 was employed for the preparation of **17c** using NIS in DMF. Yield 70%; R*_f_* = 0.46 (EtOAc); ESI-MS (*m/z*): 792.48 (M+H^+^), 514.44 (M+H^+^-ClTr), 277.76 (ClTr); ^1^Η-ΝΜR (CD_3_OD): *δ* 7.58 (d, 1H, *J* = 7.6 Hz), 7.49–7.23 (m, 13H), 7.16–7.00 (m, 8H), 6.83 (d, 1H, *J* = 8.0 Hz), 5.15 (s, 2H), 3.57 (s, 3H), 2.78 (t, 2H, *J* = 7.6 Hz), 2.57 (t, 2H, *J* = 7.6 Hz). Anal. Calcd for C_40_H_32_N_6_O_2_ClI (%): C: 60.73; H: 4.08; N: 10.62. Found (%): C: 60.66; H: 4.14; N: 10.56.

*(E)-Methyl 1-[[2′-(1H-tetrazol-5-yl)biphenyl-4-yl]methyl]imidazole-4-acrylate* (**18**). General procedure 6 was employed for the preparation of **18**. Yield 94%; R*_f_* = 0.51 (8.5:1.5 CHCl_3_:MeOH); ESI-MS (*m/z*): 387.15 (M+H^+^); ^1^Η ΝΜR (400 MHz, DMSO-*d_6_*): *δ* 8.49 (s, 1H), 7.87 (s, 1H), 7.68–7.54 (m, 4H), 7.53 (d, 1H, *J* = 15.6 Hz), 7.27 (d, 2H, *J* = 7.6 Hz), 7.13 (d, 2H, *J* = 8.0 Hz), 6.48 (d, 1H, *J* = 15.6 Hz), 5.32 (s, 2H), 3.71 (s, 3H). Anal. Calcd for C_21_H_18_N_6_O_2_∙CF_3_COOH (%): C: 55.20; H: 3.83; N: 16.79. Found (%): C: 55.11; H: 3.72; N: 16.84. 

*Methyl 1-[[2′-(1H-tetrazol-5-yl)biphenyl-4-yl]methyl]imidazole-4-propanoate* (**19**). General procedure 6 was employed for the preparation of **19**. Yield 89%; R*_f_* = 0.49 (8.5:1.5 CHCl_3_:MeOH); ESI-MS (*m/z*): 389.13 (M+H^+^); ^1^Η-ΝΜR (CD_3_OD): *δ* 7.91 (d, 1H, *J* = 7.5 Hz), 7.62-6.76 (m, 9H), 6.62 (s, 1H), 5.04 (s, 2H), 3.64 (s, 3H), 2.76 (t, 2H, *J* = 7.2 Hz), 2.56 (t, 2H, *J* = 7.2 Hz); ^13^C-NMR (CD_3_OD): *δ* 167.99, 163.90, 141.79, 141.32, 140.68, 139.38, 138.96, 137.53, 136.06, 135.08, 131.47, 130.72, 130.30, 129.66, 128.36, 127.80, 127.31, 126.52, 114.82, 50.85, 50.37, 35.75, 29.83. Anal. Calcd for C_21_H_20_N_6_O_2_∙CF_3_COOH (%): C: 54.98; H: 4.21; N: 16.73. Found (%): C: 54.86; H: 4.33; N: 16.61. 

*Methyl 5-chloro-1-[[2′-(1H-tetrazol-5-yl)biphenyl-4-yl]methyl]imidazole-4-propanoate* (**20a**). General procedure 6 was employed for the preparation of **20a**. Yield 95%; R*_f_* = 0.53 (8.5:1.5 CHCl_3_:MeOH); ESI-MS (*m/z*): 423.92 (M+H^+^); ^1^Η-ΝΜR (CD_3_OD): *δ* 8.30 (s, 1H), 7.71-7.60 (m, 2H), 7.23–7.18 (m, 4H), 5.32 (s, 2H), 3.66 (s, 3H), 2.92 (t, 2H, *J* = 7.2 Hz), 2.70 (t, 2H, *J* = 7.2 Hz). Anal. Calcd for C_21_H_19_N_6_O_2_Cl∙CF_3_COOH (%): C: 51.45; H: 3.75; N: 15.65. Found (%): C: 51.37; H: 3.83; N: 16.51. 

*Methyl 5-bromo-1-[[2′-(1H-tetrazol-5-yl)biphenyl-4-yl]methyl]imidazole-4-propanoate* (**20b**). General procedure 6 was employed for the preparation of **20b**. Yield 93%; R*_f_* = 0.52 (8.5:1.5 CHCl_3_:MeOH); ESI-MS (*m/z*): 468.39 (M+H^+^); ^1^Η-ΝΜR (CD_3_OD): *δ* 8.80 (s, 1H), 7.70–7.56 (m, 4H), 7.26–7.18 (m, 4H), 5.40 (s, 2H), 3.66 (s, 3H), 2.97 (t, 2H, *J* = 7.2 Hz), 2.73 (t, 2H, *J* = 7.2 Hz). Anal. Calcd for C_21_H_19_N_6_O_2_Br∙CF_3_COOH (%): C: 47.52; H: 3.47; N: 14.46. Found (%): C: 47.63; H: 3.36; N: 14.55. 

*Methyl 5-iodo-1-[[2′-(1H-tetrazol-5-yl)biphenyl-4-yl]methyl]imidazole-4-propanoate* (**20c**). General procedure 6 was employed for the preparation of **20c**. Yield 95%; R*_f_* = 0.53 (8.5:1.5 CHCl_3_:MeOH); ESI-MS (*m/z*): 515.38 (M+H^+^); ^1^Η-ΝΜR (CD_3_OD): *δ* 8.37 (s, 1H), 7.62–7.51 (m, 4H), 7.09 (s, 4H), 5.23 (s, 2H), 3.58 (s, 3H), 2.84 (t, 2H, *J* = 7.2 Hz), 2.60 (t, 2H, *J* = 7.2 Hz). Anal. Calcd for C_21_H_19_N_6_O_2_I∙CF_3_COOH (%): C: 43.96; H: 3.21; N: 13.37. Found (%): C: 43.87; H: 3.34; N: 13.44. 

*(E)-1-[[2′-[[N-(2-Chlorotrityl)]-1H-tetrazol-5-yl]biphenyl-4-yl]methyl]imidazole-4-acrylic aci*d (**21a**). General procedure 4 was employed for the preparation of **21a**. Yield 90%; R*_f_* = 0.38 (9:1 CHCl_3_:MeOH); ESI-MS (*m/z*): 650.17 (M+H^+^), 372.80 (M+H^+^-ClTr), 277.76 (ClTr); ^1^Η-ΝΜR (DMSO-*d_6_*): *δ* 7.81 (d, 1H, *J* = 7.6 Hz), 7.62–7.49 (m, 4H), 7.45–7.30 (m, 9H), 7.29 (d, 1H, *J* = 15.6 Hz), 7.11–7.06 (m, 4H), 6.78 (d, 4H, *J* = 7.6 Hz), 6.68 (d, 1Η, *J* = 8.0 Hz), 6.44 (d, 1H, *J* = 15.6 Hz), 5.12 (s, 2H). Anal. Calcd for C_39_H_29_N_6_O_2_Cl (%): C: 72.16; H: 4.50; N: 12.95. Found (%): C: 72.09; H: 4.58; N: 12.89.

*(E)-(5-Methyl-2-oxo-1,3-dioxol)methyl-1-[[2′-[[N-(2-chlorotrityl)]-1H-tetrazol-5-yl]biphenyl-4-yl]-methyl]imidazole-4-acrylate* (**21b**). General procedure 5 was employed for the preparation of **21b**. Yield 84%; R*_f_* = 0.52 (EtOAc); ESI-MS (*m/z*): 762.165 (M+H^+^), 484.44 (M+H^+^-ClTr), 277.76 (ClTr); ^1^Η-ΝΜR (DMSO-*d*_6_): *δ* 7.80 (s, 2H), 7.60–7.42 (m, 7H), 7.34–7.27 (m, 7H), 7.09 (s, 4H), 6.76 (s, 4H), 6.67 (s, 1H), 6.28 (d, 1H, *J* = 16.0 Hz), 5.12 (s, 2H), 5.02 (s, 2H), 2.16 (s, 3H). Anal. Calcd for C_44_H_33_N_6_O_5_Cl (%): C: 69.42; H: 4.37; N: 11.04. Found (%): C: 69.39; H: 4.32; N: 10.98.

*(E)-1-[[2′-(1H-Tetrazol-5-yl)biphenyl-4-yl]methyl]imidazole-4-acrylic acid* (**22a**). General procedure 6 was employed for the preparation of **22a**. Yield 95%; R*_f_* = 0.38 (4:1:1 *n*-butanol:acetic acid:H_2_O); ESI-MS (*m/z*): 373.44 (M+H^+^); ^1^Η-ΝΜR (DMSO-*d_6_*): *δ* 8.56 (s, 1H), 7.84 (s, 1H), 7.66 (s, 2H), 7.58 (s, 1H), 7.52 (s, 1H), 7.42 (d, 1H, *J* = 16.0 Hz), 7.26 (s, 2H), 7.11 (s, 2H), 6.40 (d, 1H, *J* = 16.0 Hz), 5.31 (s, 2H). Anal. Calcd for C_20_H_16_N_6_O_2_∙CF_3_COOH (%): C: 54.32; H: 3.52; N: 17.28. Found (%): C: 54.45; H: 3.41; N: 17.39.

*(E)-(5-Methyl-2-oxo-1,3-dioxol)methyl-1-[[2′-(1H-tetrazol-5-yl)biphenyl-4-yl]methyl]imidazole-4-acrylate* (**22b**). General procedure 6 was employed for the preparation of **22b**. Yield 94%; R*_f_* = 0.52 (8.5:1.5 CHCl_3_:MeOH); ESI-MS (*m/z*): 485.52 (M+H^+^); ^1^Η-ΝΜR (DMSO-*d_6_*): *δ* 8.10 (s, 1H), 7.76 (s, 1H), 7.65 (s, 2H), 7.57–7.51 (m, 3H), 7.24–7.20 (m, 2H), 7.11–7.07 (m, 2H), 6.38 (d, 1H, *J* = 16.0 Hz), 5.24 (s, 2H), 5.04 (s, 2H), 2.16 (s, 3H). Anal. Calcd for C_25_H_20_N_6_O_5_∙CF_3_COOH (%): C: 54.18; H: 3.54; N: 14.04. Found (%): C: 54.08; H: 3.41; N: 14.17.

*1-[[2′-[[N-(2-Chlorotrityl)]-1H-tetrazol-5-yl]biphenyl-4-yl]methyl]imidazole-4-propanoic acid* (**23a**). General procedure 4 was employed for the preparation of **23a**. Yield 93%; R*_f_* = 0.33 (9:1 CHCl_3_:MeOH); ESI-MS (*m/z*): 652.19 (M+H^+^), 374.77 (M+H^+^-ClTr), 277.77 (ClTr); ^1^Η-ΝΜR (DMSO-*d_6_*): *δ* 7.82–7.69 (m, 1H), 7.64–7.44 (m, 6H), 7.40–7.27 (m, 7H), 7.10–7.02 (m, 4H), 7.09–6.66 (m, 6H), 5.00 (s, 2H), 2.60 (t, 2H, *J* = 7.2 Hz), 2.38 (t, 2H, *J* = 7.2 Hz). Anal. Calcd for C_39_H_31_N_6_O_2_Cl (%): C: 71.94; H: 4.80; N: 12.91. Found (%): C: 71.87; H: 4.86; N: 12.8**7.**

*(5-Μethyl-2-oxo-1,3-dioxol)methyl-1-[[2′-[[N-(2-chlorotrityl)]-1H-tetrazol-5-yl]biphenyl-4-yl]-methyl]imidazole-4-propanoate* (**23b**). General procedure 5 was employed for the preparation of **23b**. Yield 73%; R*_f_* = 0.45 (9:1 CHCl_3_:MeOH); ESI-MS (*m/z*): 764.21 (M+H^+^), 486.56 (M+H^+^-ClTr), 277.76 (ClTr); ^1^Η-ΝΜR (CDCl_3_): *δ* 7.96 (d, 1H, *J* = 7.2 Hz), 7.49–7.31 (m, 9H), 7.22–7.18 (m, 5H), 7.13 (d, 2H, *J* = 8.0 Hz), 6.92-6.84 (m, 5H), 6.82 (s, 1H), 6.72 (d, 1H, *J* = 8.0 Hz), 4.88 (s, 2H), 4.81 (s, 2H), 2.83 (t, 2H, *J* = 7.2 Hz), 2.68 (t, 2H, *J* = 7.2 Hz), 2.16 (s, 3H). Anal. Calcd for C_44_H_35_N_6_O_5_Cl (%): C: 69.24; H: 4.62; N: 11.01. Found (%): C: 69.19; H: 4.67; N: 11.08. 

*1-[[2′-(1H-Tetrazol-5-yl)biphenyl-4-yl]methyl]imidazole-4-propanoic acid* (**24a**). General procedure 6 was employed for the preparation of **24a**. Yield 92%; R*_f_* = 0.33 (4:1:1 *n*-butanol:acetic acid:H_2_O); ESI-MS (*m/z*): 375.41 (M+H^+^); ^1^Η-ΝΜR (CD_3_OD): *δ* 8.89 (s, 1H), 7.71–7.67 (m, 2Η), 7.60–7.56 (m, 2H), 7.39 (s, 1H), 7.35 (d, 2H, *J* = 7.2 Hz), 7.20 (d, 2H, *J* = 7.2 Hz), 5.50 (s, 2H), 2.98 (t, 2H *J* = 7.2 Hz), 2.71 (t, 2H, *J* = 7.2 Hz). Anal. Calcd for C_20_H_18_N_6_O_2_∙CF_3_COOH (%): C: 54.10; H: 3.92; N: 17.21. Found (%): C: 54.17; H: 3.81; N: 17.32.

*(5-Methyl-2-oxo-1,3-dioxol)methyl-1-[[2′-(1H-tetrazol-5-yl)biphenyl-4-yl]methyl]imidazole-4-propanoate* (**24b**). General procedure 6 was employed for the preparation of **24b**. Yield 87%; R*_f_* = 0.49 (8.5:1.5 CHCl_3_:MeOH); ESI-MS (*m/z*): 487.56 (M+H^+^); ^1^Η-ΝΜR (DMSO-*d_6_*): *δ* 9.06 (s, 1H), 7.67–7.12 (m, 9H), 5.35 (s, 2H), 4.94 (s, 2H), 2.87 (t, 2H, *J* = 7.2 Hz), 2.74 (t, 2H, *J* = 7.2 Hz), 2.12 (s, 3H). Anal. Calcd for C_25_H_22_N_6_O_5_∙CF_3_COOH (%): C: 54.00; H: 3.86; N: 13.99. Found (%): C: 53.91; H: 3.91; N: 14.11.

*5-Chloro-1-[[2′-[[N-(2-chlorotrityl)]-1H-tetrazol-5-yl]biphenyl-4-yl]methyl]imidazole-4-propanoic acid* (**25a**). General procedure 4 was employed for the preparation of **25a**. Yield 94%; R*_f_* = 0.39 (9:1 CHCl_3_:MeOH); ESI-MS (*m/z*): 686.66 (M+H^+^), 408.92 (M+H^+^-ClTr), 277.81 (ClTr); ^1^Η-ΝΜR (CDCl_3_): *δ* 7.98–7.94 (m, 1H), 7.48-7.08 (m, 15H), 6.85 (d, 6H, *J* = 7.6 Hz), 6.71 (d, 1H, *J* = 8.0 Hz), 4.84 (s, 2H), 2.79 (bs, 2H), 2.58 (bs, 2H). Anal. Calcd for C_39_H_30_N_6_O_2_Cl_2_ (%): C: 68.31; H: 4.41; N: 12.26. Found (%): C: 68.08; H: 4.53; N: 12.57.

*(5-Methyl-2-oxo-1,3-dioxol)methyl-5-chloro-1-[[2΄-[[N-(2-chlorotrityl)]-1H-tetrazol-5-yl]biphenyl-4-yl]methyl]imidazole-4-propanoate* (**25b**). General procedure 1 was employed for the preparation of **7**. Yield 82%; R*_f_* = 0.50 (EtOAc); ESI-MS (*m/z*): 798.72 (M+H^+^), 521.03 (M+H^+^-ClTr), 277.78 (ClTr); ^1^Η-ΝΜR (CDCl_3_): *δ* 7.95 (d, 1Η, *J* = 7.2 Hz), 7.48–7.34 (m, 6H), 7.34–7.32 (m, 5H), 7.23 (m, 5H), 7.11 (d, 2H, *J* = 8.0 Hz), 6.91–6.86 (m, 8H), 4.93 (s, 2H), 4.83 (s, 2H), 2.87 (t, 2H, *J* = 7.2 Hz), 2.74 (t, 2H, *J* = 7.2 Hz), 2.15 (s, 3H). Anal. Calcd for C_44_H_34_N_6_O_5_Cl_2_ (%): C: 66.25; H: 4.30; N: 10.54. Found (%): C: 66.24; H: 4.18; N: 10.33.

*5-Bromo-1-[[2′-[[N-(2-chlorotrityl)]-1H-tetrazol-5-yl]biphenyl-4-yl]methyl]imidazole-4-propanoic acid* (**25c**). General procedure 4 was employed for the preparation of **25c**. Yield 91%; R*_f_* = 0.37 (9:1, CHCl_3_:MeOH); ESI-MS (*m/z*): 731.11 (M+H^+^), 453.36 (M+H^+^-ClTr), 277.76 (ClTr); ^1^Η-ΝΜR (CDCl_3_): *δ* 8.04 (d, 1H, *J* = 7.6 Hz), 7.67–7.28 (m, 15H), 7.09 (d, 2H, *J* = 8.0 Hz), 6.97 (d, 4H, *J* = 7.6 Hz), 6.87 (d, 1H, *J* = 8.0 Hz), 5.12 (s, 1H), 2.96–2.92 (m, 2H), 2.81–2.78 (m, 2H). Anal. Calcd for C_39_H_30_N_6_O_2_ClBr (%): C: 64.16; H: 4.14; N: 11.51. Found (%): C: 64.11; H: 4.21; N: 11.44.

*(5-Methyl-2-oxo-1,3-dioxol)methyl-5-bromo-1-[[2′-[[N-(2-chlorotrityl)]-1H-tetrazol-5-yl]biphenyl-4-yl]methyl]imidazole-4-propanoate* (**25d**). General procedure 5 was employed for the preparation of **25d**. Yield 72%; R*_f_* = 0.48 (EtOAc); ESI-MS (*m/z*): 843.18 (M+H^+^), 565.43 (M+H^+^-ClTr), 277.78 (ClTr); ^1^Η-ΝΜR (CDCl_3_): *δ* 8.03 (d, 1H, *J* = 7.2 Hz), 7.65–7.33 (m, 13H), 7.24 (d, 2H, *J* = 7.6 Hz), 6.99 (m, 4H), 6.87 (d, 1H, *J* = 7.6 Hz), 5.07 (s, 2H), 4.93 (s, 2H), 2.94 (t, 2H, *J* = 6.4 Hz), 2.82 (t, 2H, *J* = 6.4 Hz), 2.24 (s, 3H). Anal. Calcd for C_44_H_34_N_6_O_5_ClBr (%): C: 62.75; H: 4.07; N: 9.98. Found (%): C: 62.68; H: 4.15; N: 9.91.

*5-Iodo-1-[[2′-[[N-(2-chlorotrityl)]-1H-tetrazol-5-yl]biphenyl-4-yl]methyl]imidazole-4-propanoic acid* (**25e**). General procedure 4 was employed for the preparation of **25e**. Yield 95%; R*_f_* = 0.37 (9:1 CHCl_3_:MeOH); ESI-MS (*m/z*): 778.13 (M+H^+^), 500.35 (M+H^+^-ClTr), 277.76 (ClTr); ^1^Η-ΝΜR (CDCl_3_): *δ* 7.97 (d, 1H, *J* = 7.6 Hz), 7.72 (s, 1H), 7.49–7.17 (m, 15H), 6.93 (d, 2H, *J* = 7.6 Hz ), 6.88 (d, 4H, *J* = 7.6 Hz), 6.73 (d, 1H, *J* = 7.2 Hz), 4.98 (s, 2H), 2.88 (t, 2H, *J* = 7.2 Hz), 2.73 (t, 2H, *J* = 7.2 Hz). Anal. Calcd for C_39_H_30_N_6_O_2_ClI (%): C: 60.28; H: 3.89; N: 10.82. Found (%): C: 60.21; H: 3.96; N: 10.73.

*(5-Methyl-2-oxo-1,3-dioxol)methyl-5-iodo-1-[[2′-[[N-(2-chlorotrityl)]-1H-tetrazol-5-yl]biphenyl-4-yl]methyl]imidazole-4-propanoate* (**25f**). General procedure 5 was employed for the preparation of **25f**. Yield 74%; R*_f_* = 0.51 (EtOAc); ESI-MS (*m/z*): 890.21 (M+H^+^), 612.45 (M+H^+^-ClTr), 276.88 (ClTr); ^1^Η-ΝΜR (CDCl_3_): *δ* 7.77 (d, 1H, *J* = 7.6 Hz), 7.45–7.22 (m, 8Η), 7.15–6.80 (m, 14H), 4.86 (s, 2H), 4.71 (s, 2H), 2.71 (t, 2H, *J* = 7.2 Hz), 2.59 (t, 2H, *J* = 7.2 Hz), 2.02 (s, 3H); Anal. Calcd for C_44_H_34_N_6_O_5_ClI (%): C: 59.44; H: 3.85; N: 9.45. Found (%): C: 59.36; H: 3.94; N: 4.37.

*5-Chloro-1-[[2′-(1H-tetrazol-5-yl)biphenyl-4-yl]methyl]imidazole-4-propanoic acid* (**26a**). General procedure 6 was employed for the preparation of **26a**. Yield 90%; R*_f_* = 0.32 (4:1:1 *n*-butanol:acetic acid:H_2_O); ESI-MS (*m/z*): 409.31 (M+H^+^); ^1^Η-ΝΜR (CD_3_OD): *δ* 8.62 (s, 1H), 7.70 (d, 2H, *J* = 7.2 Hz), 7.62–7.56 (m, 2H), 7.27 (d, 2H, *J* = 7.6 Hz), 7.19 (d, 2H, *J* = 7.6 Hz), 5.38 (s, 2H), 2.94 (t, 2H, *J* = 7.2 Hz), 2.69 (t, 2H, *J* = 7.2 Hz). Anal. Calcd for C_20_H_17_N_6_O_2_Cl∙CF_3_COOH (%): C: 50.54; H: 3.47; N: 16.07. Found (%): C: 50.43; H: 3.58; N: 16.17.

*(5-Methyl-2-oxo-1,3-dioxol)methyl-5-chloro-1-[[2′-(1H-tetrazol-5-yl)biphenyl-4-yl]methyl]imidazole-4-propanoate* (**26b**). General procedure 6 was employed for the preparation of **26b**. Yield 89%; R*_f_* = 0.56 (8.5:1.5 CHCl_3_:MeOH); ESI-MS (*m/z*): 522.00 (M+H^+^); ^1^Η-ΝΜR (DMSO-*d_6_*): *δ* 8.24 (s, 1H), 7.71–7.54 (m, 4H), 7.17-7.08 (m, 4H), 5.24 (s, 2H), 4.92 (s, 2H), 2.76 (t, 2H, *J* = 7.6 Hz), 2.69 (t, 2H, *J* = 7.6 Hz), 2.13 (s, 3H). Anal. Calcd for C_25_H_21_N_6_O_5_Cl∙CF_3_COOH (%): C: 51.07; H: 3.49; N: 13.24. Found (%): C: 51.13; H: 3.41; N: 13.35.

*5-Bromo-1-[[2′-(1H-tetrazol-5-yl)biphenyl-4-yl]methyl]imidazole-4-propanoic acid* (**26c**). General procedure 6 was employed for the preparation of **26c**. Yield 93%; R*_f_* = 0.31 (4:1:1 *n*-butanol:acetic acid:H_2_O); ESI-MS (*m/z*): 454.37 (M+H^+^); ^1^Η-ΝΜR (CD_3_OD): *δ* 7.67–7.55 (m, 5H), 7.12 (s, 4H), 5.21 (s, 2H), 2.83 (t, 2H, *J* = 7.2 Hz), 2.60 (t, 2H, *J* = 7.2 Hz). Anal. Calcd for C_20_H_17_N_6_O_2_Br∙CF_3_COOH (%): C: 46.58; H: 3.20; N: 14.81. Found (%): C: 46.63; H: 3.32; N: 14.69.

*(5-Methyl-2-oxo-1,3-dioxol)methyl-5-bromo-1-[[2′-(1H-tetrazol-5-yl)biphenyl-4-yl]methyl]imidazole-4-propanoate* (**26d**). General procedure 6 was employed for the preparation of **26d**. Yield 91%; R*_f_* = 0.55 (8.5:1.5 CHCl_3_:MeOH); ESI-MS (*m/z*): 566.45 (M+H^+^); ^1^Η-ΝΜR (DMSO-*d_6_*): *δ* 7.91 (s, 1H), 7.67–7.64 (m, 2H), 7.58–7.53 (m, 2H), 7.09 (s, 4H), 5.17 (s, 2H), 4.92 (s, 2H), 2.72–2.63 (m, 4H), 2.13 (s, 3H). Anal. Calcd for C_25_H_21_N_6_O_5_Br∙CF_3_COOH (%): C: 47.73; H: 3.26; N: 12.37. Found (%): C: 47.83; H: 3.34; N: 13.27.

*5-Iodo-1-[[2′-(1H-tetrazol-5-yl)biphenyl-4-yl]methyl]imidazole-4-propanoic acid* (**26e**). General procedure 6 was employed for the preparation of **26e**. Yield 91%, R*_f_* = 0.29 (4:1:1 *n*-butanol:acetic acid:H_2_O); ESI-MS (*m/z*): 501.38 (M+H^+^); ^1^Η-ΝΜR (CD_3_OD): *δ* 8.79 (s, 1H), 7.68–7.64 (m, 2H), 7.56–7.31 (m, 4H), 7.19 (d, 2H, *J* = 8.0 Hz), 5.35 (s, 2H), 2.95 (t, 2H, *J* = 7.2 Hz), 2.68 (t, 2H, *J* = 7.2 Hz). Anal. Calcd for C_20_H_17_N_6_O_2_I∙CF_3_COOH (%): C: 43.01; H: 2.95; N: 13.68. Found (%): C: 43.13; H: 2.87; N: 13.79.

*(5-Methyl-2-oxo-1,3-dioxol)methyl-5-iodo-1-[[2′-(1H-tetrazol-5-yl)biphenyl-4-yl]methyl]imidazole-4-propanoate* (**26f**). General procedure 6 was employed for the preparation of **26f**. Yield 90%; R*_f_* = 0.56 (8.5:1.5 CHCl_3_:MeOH); ESI-MS (*m/z*): 613.44 (M+H^+^); ^1^Η-ΝΜR (CD_3_OD): *δ* 8.00 (s, 1H), 7.66–7.56 (m, 4H), 7.15 (bs, 4Η), 5.22 (s, 2H), 4.88 (s, 2H), 2.86 (t, 2H, *J* = 7.2 Hz), 2.70 (t, 2H, *J* = 7.2 Hz), 2.16 (s, 3H). Anal. Calcd for C_25_H_21_N_6_O_5_I∙CF_3_COOH (%): C: 44.64; H: 3.05; N: 11.57. Found (%): C: 44.72; H: 3.14; N: 11.65.

#### 3.2.9. Synthesis of 1,5-Disubstituted Benzyl Analogues **30**, **32**

*(E)-Methyl 3-[1-[(2-(trimethylsilyl)ethoxy)methyl]-1H-imidazole-4-yl]acrylate* (**27**). To a solution of (*E*)-urocanic methyl ester (**6**) (0.61 g, 4.03 mmol) in dry DMF (15.0 mL) under argon atmosphere at 0 °C was added dry NaH (powdered 95%, 0.12 g, 4.84 mmol) and the suspension was left at the same temperature for 15 min. Then, SEM-Cl (0.68 mL, 4.84 mmol) was added in three portions and the reaction mixture was allowed to warm to rt for 4 h. The reaction was quenched with 0.5 N NaOH (15 mL) and extracted with CH_2_Cl_2_ (3 × 20 mL). The combined organic phases were washed with brine (× 2), dried (Na_2_SO_4_) and concentrated *in vacuo*. Purification by flash column chromatography (7:3 EtOAc:hexanes) afforded **27**. Yield 78%; R*_f_* = 0.35 (8:2 EtOAc:hexanes); ESI-MS (*m/z*): 283.29 (M+H^+^); ^1^Η-ΝΜR (CDCl_3_): *δ* 8.58 (s, 1H), 7.52 (d, 1H, *J* = 16.0 Hz), 7.35 (s, 1H), 6.68 (d, 1H, *J* = 16.0 Hz), 5.42 (s, 2H), 3.82 (s, 3H), 3.58 (t, 2H, *J* = 8.0 Hz), 0.96 (t, 2H, *J* = 8.0 Hz), 0.01 (s, 9H); ^13^C-NMR (CDCl_3_): *δ* 166.64, 137.98, 134.48, 130.47, 121.21, 120.59, 50.85, 76.79, 68.14, 51.95, 18.22, 1.44. Anal. Calcd for C_13_H_22_N_2_O_3_Si (%): C: 55.29; H: 7.85; N:9.92. Found (%): C:55.36; H:7.72; N:9.87.

*Methyl 3-[1-[(2-(trimethylsilyl)ethoxy)methyl]-1H-imidazole-4-yl]propanoate* (**28**). General procedure 2 was employed for the preparation of **28**. Yield 91%; R*_f_* = 0.44 (9:1 CHCl_3_:MeOH); ESI-MS (*m/z*): 284.99 (M+H^+^); ^1^Η-ΝΜR (CDCl_3_): *δ* 7.91 (s, 1H), 6.88 (s, 1H), 5.25 (s, 2H), 3.67 (s, 3H), 3.50 (t, 2H, *J* = 8.0 Hz), 3.00 (t, 2H, *J* = 7.2 Hz), 2.72 (t, 2H, *J* = 7.2 Hz), 0.91 (t, 2H, *J* = 8.0 Hz), 0.01 (s, 9H); ^13^C-NMR (CDCl_3_): *δ* 173.13, 136.14, 116.19, 76.88, 67.08, 120.59, 51.65, 33.27, 22.27, 17.89, 1.44. Anal. Calcd for C_13_H_24_N_2_O_3_Si (%): C:54.90; H:8.51; N:9.85. Found (%): C:54.81; H:8.61; N:9.74.

*Methyl* *1-[[2′-[[N-(2-chlorotrityl)]-1H-tetrazol-5-yl]biphenyl-4-yl]methyl]-3-[(2-(trimethylsilyl)-ethoxy)methyl]imidazolium-5-propanoate bromide* (**29**). To a stirred solution of **28** (2.0 g, 7.03 mmol) in dry CH_2_Cl_2_ (20.0 mL) under argon was added the alkylating agent **4** (4.31 g, 7.73 mmol) in one portion and the resulting mixture was heated under reflux for 3 h. Upon completion (disappearance of starting material confirmed by RP-HPLC), the solvent was concentrated, followed by chromatographic purification (97:3 CHCl_3_:MeOH) to afford **29** as a white powder. Yield 81%; R*_f_* = 0.40 (9:1 CHCl_3_:MeOH); ESI-MS (*m/z*): 796.19 (M^+^-Br); ^1^Η-ΝΜR (CDCl_3_): *δ* 7.93 (dd, 1H, *J* = 1.6, 7.6 Hz), 7.52–7.46 (m, 3H), 7.40–7.13 (m, 13H), 7.11 (s, 1H), 6.94 (d, 6H, *J* = 7.6 Hz), 5.67 (s, 2H), 5.46 (s, 2H), 3.75–3.64 (m, 5H), 2.75 (t, 2H, *J* = 7.2 Hz), 2.47 (t, 2H, *J* = 7.2 Hz), 0.94 (t, 2H, *J* = 8.0 Ηz), 0.002 (s, 9H). Anal. Calcd for C_46_H_48_N_6_O_3_SiBr (%): C:63.04; H:5.52; N:9.59. Found (%): C:62.93; H:5.57; N:9.51.

*Methyl 1-[[2′-(1H-tetrazol-5-yl)biphenyl-4-yl]methyl]-3-[(2-(trimethylsilyl)ethoxy)methyl]imidazole-5-propanoate* (**30**). General procedure 6 was employed for the preparation of **30**. Yield 90%; R*_f_* = 0.53 (8.5:1.5 CHCl_3_:MeOH); ESI-MS (*m/z*): 389.51 (M+H^+^); ^1^Η-ΝΜR (DMSO-*d_6_*): *δ* 9.13 (s, 1H), 7.68 (d, 1H, *J* = 8.0 Hz), 7.59 (d, 1H, *J* = 7.6 Hz), 7.54 (d, 1H, *J* = 7.6 Hz), 7.50 (s, 1H), 7.21 (d, 2H, *J* = 8.0 Hz), 7.14 (d, 1H, *J* = 8.0 Hz), 5.48 (s, 2H), 3.60 (s, 3H), 2.77 (t, 2H, *J* = 7.2 Hz), 2.64 (t, 2H, *J* = 7.2 Hz). Anal. Calcd for C_21_H_20_N_6_O_2_∙CF_3_COOH (%): C:54.98; H:4.21; N:16.73. Found (%): C:54.91; H:4.13; N:16.61.

*Methyl 2-hydroxymethyl-1-[[2′-[[N-(2-chlorotrityl)]-1H-tetrazol-5-yl]biphenyl-4-yl]methyl]-3-[(2-(trimethylsilyl)ethoxy)methyl]imidazolium-5-propanoate bromide* (**31**). In a sealed tube were sequentially added **29** (2.0 g, 2.38 mmol), DMF (1.0 mL), 37% formalin (2.65 mL, 35.63 mmol) and diisopropylethylamine (2.02 mL, 11.90 mmol). The resulting mixure was stirred at 85 °C until HPLC showed no starting material left (*ca.* 1 h). Then, the mixture was quenched with 5% aqueous citric acid (10 mL), extracted with CH_2_Cl_2_ and the combined organic phases were washed with brine, dried (Na_2_SO_4_), filtered and concentrated *in vacuo*. Purification by flash column chromatography (96:4 CHCl_3_:MeOH) afforded **31**. Yield 91%; R*_f_* = 0.28 (9:1 CHCl_3_:MeOH); ESI-MS (*m/z*): 826.32 (M^+^-Br); ^1^Η-ΝΜR (CDCl_3_): *δ* 7.95 (d, 1H, *J* = 6.8 Hz), 7.52–7.42 (m, 3H), 7.40–7.26 (m, 9H, ), 7.19 (s, 1H), 7.15 (d, 2H, *J* = 8.0 Hz), 6.94–6.89 (m, 8H), 5.76 (s, 2H), 5.52 (s, 2H), 4.72 (s, 2H), 3.70–3.62 (m, 5H), 2.76 (t, 2H, *J* = 6.8 Hz), 2.54 (t, 2H, *J* = 6.8 Hz), 0.95 (t, 2H, *J* = 8.0 Ηz), 0.001 (s, 9H). Anal. Calcd for C_47_H_50_N_6_O_4_SiBr (%): C:62.28; H:5.56; N:9.27. Found (%): C:62.37; H:5.43; N:9.19.

*Methyl 2-hydroxymethyl-1-[[2′-(1H-tetrazol-5-yl)biphenyl-4-yl]methyl]-3-[(2-(trimethylsilyl)ethoxy)-methyl]imidazole-5-propanoate* (**32**). General procedure 6 was employed for the preparation of **32**. Yield 88%; R*_f_* = 0.32 (8:2 CHCl_3_:MeOH); ESI-MS (*m/z*): 419.56 (M+H^+^); ^1^Η-ΝΜR (DMSO-*d*_6_): *δ* 7.67 (dd, 2H, *J* = 2.0, 7.6 Hz), 7.59 (d, 1H, *J* = 7.6 Hz), 7.52 (d, 1H, *J* = 7.6 Hz), 7.44 (s, 1H), 7.12 (s, 4H), 6.26 (bs, 1H), 5.45 (s, 2H), 4.74 (s, 2H), 3.58 (s, 3H), 2.71 (t, 2H, *J* = 6.8 Hz), 2.61 (t, 2H, *J* = 6.8 Hz). Anal. Calcd for C_22_H_22_N_6_O_3_∙CF_3_COOH (%): C:54.14; H:4.35; N:15.78. Found (%): C:54.23; H:4.25; N:15.86.

### 3.3. Pharmacological Evaluation

#### 3.3.1. Radioligand Binding Assay

Radioligand binding assay was performed as previously described [20].

### 3.4. Docking Studies

The 3D model of the AT1 receptor used in our docking studies was kindly provided by Tuccinardi*et al.* [29]. The construction of this model is based on X-ray bovine rhodopsin structure, molecular procedure and available site-directed mutagenesis data [30]. Molecular Docking studies were performed using Glide extra precision (XP) implemented Induced Fit Docking (IFD) protocol (v 5.0) [31,32,33] docking programs under the Linux operating system. The active site was defined by 20 Å inner cubic grid box, centered on the point that is the center of mass of residues Lys199 and His256. The IFD protocol under the Schrodinger molecular modeling package was used in order to eliminate clashes between receptor and ligand atoms and for the receptor to gain partial flexibility to the receptor. Before the docking simulations, the complexes were submitted to the protein preparation module of Schrodinger. Ligands were constructed using the Schrodinger’s Maestro module and then geometry optimization was performed for these ligands using Polak-Ribiere conjugate gradient (PRCG) minimization (0.0001 kJÅ^−1^ mol^−1^ convergence criteria). Protonation states of residues were created using LigPrep and Protein Preparation modules under the Schrodinger package at neutral pH. IFD uses the Glide docking program to account the ligand flexibility and the refinement module and the Prime (v.1.6) program [32,33] to account for flexibility of the receptor. Schrodinger’s IFD protocol model uses the following steps (the description below is taken from the IFD user manual): (i) Constrained minimization of the receptor with an RMSD cutoff of 0.18 Å. (ii) Initial Glide docking of each ligand using soft potentials (0.5 van der Waals radii scaling of non-polar atoms of ligands and receptor using partial charge cutoff of 0.15). (iii) Derived docking poses were refined using the Prime Induced Fit module of Schrodinger. Residues within 5.0 Å of ligand poses were minimized in order to form suitable conformations of poses at the active site of the receptor. (iv) Glide re-docking of each protein–ligand complex.

## 4. Conclusions

In the present study, we have demonstrated an efficient and convenient strategy for the syntheses of a series of *N*-benzyl and *N*-biphenylmethyl imidazole derivatives substituted either at the *N*-1 or *N*-3 positions of (*E*)-urocanic acid. A facile and clean methodology with a few-step synthetic protocol in high yields has been developed. Biological evaluation of the synthesized analogues concerning their binding affinity for the AT1 receptor revealed that certain analogues (compounds **12** and **18**) are moderate inhibitors. In particular, the methyl acrylate analogue **18** which bears the biphenylmethyl moiety, showed relevant higher activity compared to the others. In addition, the lack of a lipophilic alkyl chain may also explain the lower activity of **18** which seems to be critical for binding affinity, compared to losartan. Docking results showed that flexibility of these molecules is an important factor that governs their drug activity. The synthesized analogues adopt different orientations in the active site as indicated by the docking studies. It is reasonable to assume that the studied molecules first adopt the most comfortable conformation and orientation and show the highest scoring when these are approaching the receptor. Such a hypothesis can explain the experimental data which have indicated the poor activity of the studied molecules. The propensity of some molecules to adopt the appropriate orientation and thus exert all favored but not maximal interactions can explain their moderate activity.

## References

[1] Naik P., Murumkar P., Giridhar R., Yadav M.R. (2010). Angiotensin II receptor type 1 (AT_1_) selective nonpeptidic antagonists—A perspective. Bioorg. Med. Chem..

[2] Burnier M. (2001). Angiotensin II Type 1 Receptor Blockers. Circulation.

[3] Burnier M., Brunner H.R. (2000). Angiotensin II receptor antagonists. Lancet.

[4] Cappelli A., Mohr G.P., Gallelli A., Rizzo M., Anzini M., Vomero S., Mennuni L., Ferrari F., Makovec F., Menziani M.C. (2004). Design, synthesis, structural studies, biological evaluation, and computational simulations of novel potent AT1 angiotensin II receptor antagonists based on the 4-phenylquinoline structure. J. Med. Chem..

[5] Cappelli A., Nannicini C., Giuliani G., Valenti S., Mohr G.P., Anzini M., Mennuni L., Ferrari F., Caselli G., Giordani A. (2008). Design, synthesis, and biological evaluation of AT1 angiotensin II receptor antagonists based on the pyrazolo[3,4-b]pyridine and related heteroaromatic bicyclic systems. J. Med. Chem..

[6] Kubo K., Kohara Y., Imamiya E., Sugiura Y., Inada Y., Furukawa Y., Nishikawa K., Naka T. (1993). Nonpeptide angiotensin II receptor antagonists. Synthesis and biological activity of benzimidazolecarboxylic acids. J. Med. Chem..

[7] Duncia J.V., Chiu A.T., Carini D.J., Gregory G.B., Johnson A.L., Price W.A., Wells G.J., Wong P.C., Calabrese J.C., Timmermans P.B.M.W.M. (1990). The discovery of potent nonpeptide angiotensinII receptor antagonists: A new class of potent antihypertensives. J. Med. Chem..

[8] Carini D.J., Duncia J.V., Aldrich P.E., Chiu A.T., Johnson A.L., Pierce M.E., Price W.A., Santella J.B., Wells G.J., Wexler R.R. (1991). Nonpeptide angiotensin II receptor antagonists: The discovery of a series of *N*-(biphenylylmethyl)imidazoles as potent, orally active antihypertensives. J. Med. Chem..

[9] Yanagisawa H., Ameniya Y., Kanazaki T., Shimoji Y., Fujimoto K., Kitahara Y.M., Sada T., Mizuno M., Ikeda M., Miyamoto S. (1996). Nonpeptide angiotensin II receptor antagonists: Synthesis, biological activities, and structure−activity relationships of imidazole-5-carboxylic acids bearing alkyl, alkenyl, and hydroxyalkyl substituents at the 4-position and their related compounds. J. Med. Chem..

[10] Kohara Y., Kubo K., Imamiya E., Wada T., Inada Y., Naka T. (1996). Synthesis and angiotensin II receptor antagonistic activities of benzimidazole derivatives bearing acidic heterocycles as novel tetrazole bioisosteres. J. Med. Chem..

[11] Baker W.L., White W.B. (2011). Azilsartan medoxomil: A new angiotensin II receptor antagonist for treatment of hypertension. Ann. Pharmacother..

[12] Miura S., Karnik S.S., Saku K. (2011). Significance of pigment spithelium-derived factor levels with angiotensin II type 1 receptor blokers in patients with successful colonary stent implantation. J. Renin. Angiotensin. Aldosterone. Syst..

[13] Herr R.J. (2002). 5-Substituted-1H-tetrazoles as carboxylic acid isosteres: Medicinal chemistry and synthetic methods. Bioorg. Med. Chem..

[14] Deprez P., Guillaume J., Becker R., Corbier A., Didierlaurent S., Fortin M., Frechet D., Hamon G., Heckmann B., Heitch H. (1995). Sulfonylureas and sulfonylcarbamates as new non-tetrazole angiotensin II receptor antagonists. Discovery of a highly potent orally active (Imidazolylbiphenylyl)sulfonylurea (HR 720). J. Med. Chem..

[15] Ismail M.A.H., Barker S., Abou El Ella D.A., Abouzid K.A.M., Toubar R.A., Todd M.H. (2006). Design and synthesis of new tetrazolyl- and carboxy-biphenylylmethyl-quinazolin-4-one derivatives as angiotensin II AT1 Receptor Antagonists. J. Med. Chem..

[16] Xu J.Y., Zeng Y., Ran Q., Wie Z., Bi Y., He Q.H., Wang Q.J., Hu S., Zhang J., Tang M.Y. (2007). Synthesis and biological activity of 2-alkylbenzimidazoles bearing a *N*-phenylpyrrole moiety as novel angiotensin II AT1 receptor antagonists. Bioorg. Med. Chem. Lett..

[17] Kaur N., Kaur A., Bansal Y., Shah D.V., Bansal G., Singh M. (2008). Design, synthesis, and evaluation of 5-sulfamoyl benzimidazole derivatives as novel angiotensin II receptor antagonists. Bioorg. Med. Chem..

[18] Agelis G., Resvani A., Durdagi S., Spyridaki K., Tumova T., Slaninova J., Giannopoulos P., Vlahakos D., Liapakis G., Mavromoustakos Τ. (2012). The discovery of new potent non-peptide angiotensin II AT1 receptor blockers: A concise synthesis, molecular docking studies and biological evaluation of *N*-substituted 5-butylimidazole derivatives. Eur. J. Med. Chem..

[19] Agelis G., Roumelioti P., Resvani A., Durdagi S., Androutsou M.E., Kelaidonis K., Mavromoustakos Τ., Matsoukas J. (2010). An efficient synthesis of a rationally designed 1,5 disubstituted imidazole AT1 angiotensin II receptor antagonist: Reorientation of imidazole pharmacophore groups in losartan reserves high receptor affinity and confirms docking studies. J. Comput.-Aided. Mol. Des..

[20] Agelis G., Resvani A., Matsoukas M.T., Tselios T., Kelaidonis K., Kalavrizioti D., Vlahakos D., Matsoukas J. (2011). Towards non-peptide ANG II AT1 receptor antagonists based on urocanic acid: rational design, synthesis and biological evaluation. Amino Acids.

[21] Weinstock J., Keenan R.M., Samanen J., Hempel J., Finkelstein J.A., Franz R.G., Gaitanopoulos D.E., Girard G.R., Gleason J.G., Hill D.T. (1991). 1-(Carboxybenzyl)imidazole-5-acrylic acids: Potent and selective angiotensin II receptor antagonists. J. Med. Chem..

[22] Keenan R.M., Weinstock J., Finkelstein J.A., Franz R.G., Gaitanopoulos D.E., Girard G.R., Hill D.T., Morgan T.M., Samanen J., Peishoff C.E. (1993). Potent nonpeptide angiotensin II receptor antagonists. 2. 1-(Carboxybenzyl)imidazole-5-acrylic acids. J. Med. Chem..

[23] Bovy P.R., Reitz D.B., Collins J.T., Chamberlain T.S., Olins G.M., Corpus V.M., McMahon E.G., Palomo M.A., Koepke J.P., Smits G.J. (1993). Nonpeptide angiotensin II antagonists: *N*-phenyl-1H-pyrrole derivatives are angiotensin II receptor antagonists. J. Med. Chem..

[24] Kristensen J., Lysén M., Begtrup M. (2001). Synthesis of ortho substituted arylboronic esters by *in situ* trapping of unstable lithio intermediates. Org. Lett..

[25] Amantini D., Beleggia R., Fringuelli F., Pizzo F., Vaccaro L. (2004). TBAF-catalyzed synthesis of 5-substituted 1*H*-tetrazoles under solventless conditions. J. Org. Chem..

[26] Pirrung M.C., Pei T. (2000). Synthesis of (+/−)-homohistidine. J. Org. Chem..

[27] Lipshutz B.H., Vaccaro W., Huff B. (1986). Protection of imidazoles as their β-trimethylsilylethoxymethyl (SEM) derivatives. Tetrahedron Lett..

[28] Luo G., Chen L., Dubowchik G. (2006). Regioselective Protection at *N*-2 and Derivatization at *C*-3 of Indazoles. J. Org. Chem..

[29] Tuccinardi T., Calderone V., Rapposelli S., Martinelli A. (2006). Proposal of a new binding orientation for non-peptide AT1 antagonists: Homology modeling, docking and three-dimensional quantitative structure-activity relationship analysis. J. Med. Chem..

[30] Okada T., Sugihara M., Bondar A.N., Elstner M., Entel P., Buss V. (2004). The retinal conformation and its environment in rhodopsin in light of a new 2.2 Å crystal structure. J. Mol. Biol..

[31] Friesner R.A., Murphy R.B., Repasky M.P., Frye L.L., Greenwood J.R., Halgren T.A., Sanschagrin P.C., Mainz D.T. (2006). Extra precision glide: Docking and scoring incorporating a model of hydrophobic enclosure for protein−ligand complexes. J. Med. Chem..

[32] Sherman W., Day T., Jacobson M.P., Friesner R.A., Farid R. (2006). Novel procedure for modeling ligand/receptor induced fit effects. J. Med. Chem..

[33] (2012). Small-Molecule Drug Discovery Suite 2012: Glide.

